# The Implementation of Integrated Health Information Systems – Research Studies from 7 Countries Involving the InterRAI Assessment System

**DOI:** 10.5334/ijic.6968

**Published:** 2023-02-13

**Authors:** Johanna de Almeida Mello, Nathalie IH Wellens, Kirsten Hermans, Matthieu De Stampa, Valérie Cerase, Natalie Vereker, Pálmi V. Jónsson, Harriet Finne-Soveri, Brigette Meehan, Anja Declercq

**Affiliations:** 1 LUCAS – Centre for Care Research and Consultancy, KU Leuven, Leuven, Belgium; 2 Department of Oral Health, KU Leuven, Leuven, Belgium; 3 La Source School of Nursing, University of Applied Sciences and Arts Western Switzerland (HES-SO), Lausanne, Switzerland; 4 Centre Gérontologique Départemental, Marseille, France; 5 CESP – Centre de recherche en Epidémiologie et Santé des Populations U-1018 INSERM, UPS UVSQ, France; 6 interRAI Ireland, Change and Innovation, Health Services Executive (HSE), Ireland; 7 Department of Geriatrics, Landspitali University Hospital and Faculty of Medicine, University of Iceland, Iceland; 8 Finnish Institute for Health and Welfare (THL), Helsinki, Finland; 9 Technical Advisory Services (TAS), interRAI New Zealand, New Zealand; 10 Centre for Sociological Research, CeSO, KU Leuven, Leuven, Belgium

**Keywords:** digital health assessment, integrated system, integrated care, interRAI Suite

## Abstract

**Introduction:**

In the past years, governments from several countries have shown interest in implementing integrated health information systems. The interRAI Suite of instruments fits this concept, as it is a set of standardised, evidence-based assessments, which have been validated for different care settings. The system allows the electronic transfer of information across care settings, enabling integration of care and providing support for care planning and quality monitoring. The main purpose of this research is to describe the recent implementation process of the interRAI instruments in seven countries: Belgium, Switzerland, France, Ireland, Iceland, Finland and New Zealand.

**Methods:**

The study applied a case study methodology with the focus on the implementation strategies in each country. Principal investigators gathered relevant information from multiple sources and summarised it according to specific aspects of the implementation process, comparing them across countries. The main implementation aspects are described, as well as the main advantages and barriers perceived by the users.

**Results:**

The seven case studies showed that adequate staffing, appropriate information technology, availability of hardware, professional collaboration and continuous training are perceived as important factors which can contribute to the implementation of the interRAI instruments. In addition, the use of electronic standardised assessment instruments such as the interRAI Suite provided evidence to improve decision-making and quality of care, enabling resource planning and benchmarking.

**Conclusion:**

In practice, the implementation of health information systems is a process that requires a cultural shift of policymakers and professional caregivers at all levels of health policy and service delivery. Information about the implementation process of the interRAI Suite in different countries can help investigators and policymakers to better plan this implementation. This research sheds light on the advantages and pitfalls of the implementation of the interRAI Suite of instruments and proposes approaches to overcome difficulties.

## Introduction

Care pathways of people with chronic conditions or people who need care can be very complex, as clients and family members usually have to find their way through a maze of service and care providers, often spread across different settings. In countries within the Organisation for Economic Co-operation and Development (OECD), ageing of the population and the increasing numbers of people with chronic diseases shift the focus of health care services away from acute care towards longer episodes of health care service delivery [1]. This evolution emphasises the need for integrated systems capable of addressing continuous, coordinated, and high-quality care delivery throughout people’s lifetime. In recent decades, OECD countries have introduced integrated care initiatives aimed at ensuring that individuals receive the right care, in the right place, at the right time, although existing organisational structures appear to hamper their success [1]. Integrated care between health services, social services and other care providers is based on the development of multi-disciplinary teams and/or care networks that support clients with complex needs [2]. In the OECD framework, the use of health information technology is considered a way to overcome challenges and to improve the overall performance of integrated healthcare delivery. The interRAI assessment instruments are tools which can be useful in this framework, as they are evidence-based assessment instruments, standardised and validated for different care settings, therefore, using a common language across settings and enabling an effective electronic transfer of information. The instruments are holistic, including several domains such as functional performance, cognitive functioning, psycho-social situation, health condition and diagnoses, environment and living situation, etc. They were developed and are constantly reviewed by interRAI, which is a non-profit consortium of clinicians, researchers and policy makers from over 35 countries, all with the common goal of improving the quality of life of vulnerable persons through the development of comprehensive electronic assessment systems and tools (http://www.interrai.org). [3].

The executive branch of the federal government of the United States of America (USA) commissioned the development of the first RAI “family” of instruments in 1987 [4]. The former Resident Assessment Instrument, also called the Minimum Data Set (MDS), was implemented in nursing homes in the USA in the early to mid-nineties [5]. Since then, several other instruments were developed for different care settings. The latest generation of interRAI instruments – the interRAI Suite – was first released in 2005 [6], which provides instruments with a common set of core items across settings, as well as with some specific items for specific care settings [3]. Some examples of interRAI Suite instruments are the assessment for the community setting – the interRAI Home Care instrument (interRAI HC), for the residential setting – the interRAI Long-Term Care Facilities instrument (interRAI LTCF), as well as the instrument for the acute care setting – the interRAI Acute Care (interRAI AC) and the interRAI Mental Health for community (interRAI CMH) or inpatient care (interRAI MH). The goal of the interRAI Suite is to enable essential information sharing and the transfer of data across settings. Therefore, continuity of care is enhanced, and professionals can follow the clients’ information through their care trajectories (e.g. from community care to residential care setting or to acute care setting and so on.) [7]. The interRAI Suite also provides a set of common outcomes which are useful for care planning. Some examples are the interRAI scales, validated with gold standard scales, and the CAPs (Clinical Assessment Protocols or Collaborative Action Points), which are trigger algorithms to alert caregivers of specific problem situations and provide guidelines for care planning. [8,9]. The scales and trigger algorithms are automatically generated in the electronic application when the interRAI assessment is filled out.

An OECD report from 2013 showed that the interRAI instruments enabled integrated eCare and allowed high quality data sharing. Moreover, quality indicators are available, which use aggregated data to identify the prevalence or incidence of outcomes within organisations, enabling benchmarking. To calibrate these quality measures, the indicators are risk-adjusted. These quality indicators were seen as very useful for quality improvement, as well as for public reporting and for benchmarking nationally and internationally [3].

Another type of output from the interRAI system are the Resource Utilisation Groups (RUGs), which enable the classification of clients in resource allocation groups based upon their care utilisation. Each client is allocated into a RUG group, and a case-mix index per group shows the relative use of resources (cost) of care for this client [10,11]. The RUG system has proven to be robust across countries, and is used in the United States, Canada, Iceland and Finland as a basis for funding of long-term care. Other interRAI case-mix systems have also been validated for persons receiving home care, as well as for adults receiving mental health care, for people with intellectual disabilities and for children and youth receiving mental health services [12].

In the past decades, governments of several countries have shown interest in the interRAI Suite [13,14,15,16,17,18]. In this paper, we describe the recent implementation process in seven countries: Belgium, Switzerland, France, Ireland, Iceland, Finland and New Zealand. The goal of the study is to describe the implementation process in each country and to describe and compare the advantages and pitfalls of the use of the interRAI instruments. This is useful information that can help other investigators to build strategies to implement electronic assessments in health and social care settings.

## Methods

This paper consists of a multi-site study research focusing on the implementation strategies of the interRAI instruments in seven countries. Case study research fits our research questions as we wish to explain ‘how’ countries implemented the interRAI Suite by describing the testing and the implementation process, as well as ‘why’ this instrument was chosen [19]. Case study research is an increasingly used technique and it has been identified as a stand-alone qualitative approach [20,21].

Although countries did not follow a specific implementation framework, we can fit the Implementation Mapping Framework to evaluate the implementation strategies described in this research [22]. Implementation Mapping involves five specific tasks: (1) conduct a needs assessment and identify program adopters and implementers; (2) state adoption and implementation outcomes and performance objectives, identify determinants, and create matrices of change objectives; (3) choose theoretical methods and select or design implementation strategies; (4) produce implementation protocols and materials; and (5) evaluate implementation outcomes. In this research, we concentrate in the last task, identifying advantages, disadvantages, contributing factors and barriers of the implementation of the interRAI assessment system.

## Materials

In case study research, potential data sources may include documentation, archival records, interviews, direct observations, among other types of sources [23]. In our research, research teams from each country gathered relevant information from multiple sources, such as scientific papers, reports, policy papers, working documents, constructed from the input from professional caregivers, researchers, policy makers and stakeholders. The information was then summarised according to specific aspects of the implementation processes.

## Results

The following paragraphs will list and briefly outline the steps towards implementation.

## InterRAI implementation in Belgium

In 2003, the Belgian Federal government decided that a comprehensive geriatric assessment instrument was needed in order to improve the quality of care for older persons. This instrument had to meet the following conditions:

– Be internationally validated;
– Be adaptable to different contexts;
– Give a holistic view of the older person’s situation;
– Provide the necessary input to create an adequate care plan;
– Promote collaboration in a multidisciplinary team; and
– Promote continuity of care.

Subsequently, four instruments were tested during the Interface evaluation project [24]. The interRAI Suite of instruments came out as the most suitable option by meeting all those criteria. Based on the conclusions of the Interface project, the interRAI Suite of instruments was tested as to its feasibility for implementation in the care sector. The term ‘BelRAI’ was created, which relates to the use of the interRAI instruments in Belgium. First, the instruments and manuals had to be translated into the three national languages (French, Dutch and German) and must be validated to the Belgian context of care. The complexity of the language aspects and the differences in care contexts, as well as the fragmentation of government responsibilities made the implementation more complex. Between 2006 and 2009, three instruments were piloted: the interRAI Home Care instrument (interRAI HC), the interRAI Long-Term Care Facilities instrument (interRAI LTCF) and the interRAI Acute Care (interRAI AC) [25,26,27,28,29].

From 2010 until 2017, the interRAI HC was used as a mandatory evaluation instrument for innovative home care interventions participating in a national study called Protocol 3 [30]. The interventions were considered to be innovative in the Belgian home care context because they were not part of regular care and they aimed at keeping frail older people longer at home. The interRAI HC was the main evaluation instrument in the Protocol 3 project and showed an added-value in the research and for policy making, especially as it made it possible to collect standardised and complete data about the people receiving care. Moreover, interRAI HC data was also collected in Belgium for the European project IBenC, involving 6 European countries [31].

In the period between 2012 and 2015, the interRAI Palliative Care (interRAI PC) instrument was tested in 15 Belgian nursing homes [32]. Results showed that the interRAI PC had many advantages for its use as a comprehensive assessment tool in nursing homes. However, policy makers, software providers, researchers and clinicians still need to collaborate more in order to optimize the use of the interRAI PC in nursing homes.

In 2015, a ‘BelRAI’ Screener was developed for the Belgian home care setting. This instrument consisted of five short modules in total, of which four were from the interRAI HC instrument. One module derived from the interRAI Mental Health (MH) instrument. This short screening instrument determined whether a person should receive a full interRAI HC assessment based on a validated cut off value [33]. Since June 2021, the BelRAI Screener is being used as a decision aid tool to determine whether care dependent people are entitled to extra financing for their care. Additionally, the Screener is being used by family care services to determine whether care dependent persons are entitled to a discount for family care. In 2021, a Social Supplement for the interRAI Suite has been developed to assess a client’s social context [34].

Since 2017, other interRAI instruments are being developed and/or piloted in Flanders: The Community Mental Health instrument (CMH), the Mental Health instrument (MH), as well as the corresponding Supplements (the Addictions Supplement, the Forensic Supplement, the Intellectual Disability Supplement, etc.), an instrument for Rehabilitation services, and the Screener for Children and Adolescents.

During the testing and piloting of the interRAI instruments in Belgium, as early adopters were using these instruments, systematic feedback was gathered to evaluate feasibility of scaling up the use of the assessments and to pave the way for national implementation.

In 2018, after more than 12 years of research, the Federal government has mandated the national implementation of the interRAI instruments in Belgium. Two IT-platforms were developed in order to complete the instruments: the BelRAI 2.0 (Federal platform) and the Flemish BelRAI Platform (Flemish application). These platforms are linked and all data is securely saved in a central Federal database. The applications enable the transfer of client data between health care settings and therefore ensure continuity of care and facilitate multidisciplinary collaboration. Another feature of the applications is that professional caregivers can observe the evolution of their clients’ outcomes (CAPs, scales and RUG groups) individually or in groups by using statistical software imbedded in the tool [9].

The national implementation of the interRAI instruments occurs in a stepwise way as policy makers are aware of the need for investment in hardware and network expansion in many care organisations, as well as the need for compatibility between the existing electronic patients’ records and the BelRAI applications. Additionally, all care professionals using the interRAI instruments need training. Training programs are organised according to a train-the-trainer principle, where interRAI experts from recognised interRAI training organisations train trainers, who in turn provide training to professional caregivers and other people involved.

From June 2023 onwards, the interRAI HC instrument will be mandated for Flemish home care organisations and the interRAI LTCF instrument for nursing homes, to be used for the purposes of care planning and care quality management. The implementation timing of all other interRAI instruments is yet to be determined.

## InterRAI implementation in Switzerland

The Swiss implementation followed a two-track path of upgrading the MDS-version and fully unlocking the potential of the Suite. When leveraged optimally, the interRAI mindset can become fully embedded in clinical routine processes to fuel decision making and to enable data transfer across the care continuum. The question however is how to build the road to reach that goal?

The MDS 1.0 instruments have been around for about two decades. For resource allocation in residential care, a Swiss adaptation of the MDS for nursing homes (MDS-NH) calculates a RUG-inspired case mix which is formalised in the federal law as one of three funding mechanisms. Its clinical application, the main driver of interRAI, however is underutilised in Swiss nursing homes. Home care agencies perform assessments in combination with registering clinical acts performed, mainly to inform and/or backup insurance claims (e.g. documenting care complexity and justifying why procedures took longer than the predefined average minutes). Efforts to facilitate the integration of the CAPs and scales in care planning vary considerably. In preparing the transition from the older to the recent version, a lot of attention is given to seizing the opportunity to refocus on clinical value.

To strengthen continuity and coordination of care, canton Vaud launched a policy program named coRAI (“une coordination de soins renforcée par la Suite interRAI”) to foster a common language across the healthcare system [35]. The roadmap has four main phases. In the “Preparation phase” (2014–2015), the interRAI language and digital data platforms were consolidated in a legal framework [36- Decree 800.033 dated 7.12.2016]. Awareness and detection of early decline and sharing of uniform functional data were promoted. Current assessment systems and their usage were examined. During the “Pilot phase” (2016–2018), trained staff tested several interRAI tools in routine practice in multicentre exploratory studies, with emphasis on care planning and preventive actions in both nursing homes and home care agencies [37,38]. Mixed methods research evaluated acceptability and feasibility. In a second wave (2018–2019), 13 cantons participated in research comparing national case-mix systems for resource allocation in nursing homes. A third wave (2018–2021) expanded pilots in short stay and day care, protected living, and mental health foyers [39]. In the “Policy phase” (2020–2022) several cantons collaborated to outline implementation strategies regarding the legal frameworks, cost simulations, timelines, etc. In the “Implementation phase”, the interRAI Check Up [40], in tandem with MDS-HC after stratification with the interRAI Assessment Urgency Algorithm [41], was adopted in all Vaudois public home services (2019). Also, interRAI Cognitive Performance Scale (CPS) and interRAI Activities of Daily Living Hierarchy (ADLH) scales were automatically included from the electronic hospital record into the existing Vaudois transfer forms between providers. In 2021, home care data was linked to hospitalisation databases and population registries to explore the potential for illustrating care trajectories to inform policy [42]. Next, to raise awareness pertaining to data-driven quality issues and to illustrate the potential of structured clinical routine data, Switzerland participated in an international benchmark that was shared with clinical and political decision makers [43].

In the meantime, the national agencies prepared the nationwide upgrade of the interRAI LTCF, interRAI HC and interRAI CMH (2019–2022) as mentioned above. This switch was beyond changing the item coding and involved software licensing to 15 software vendors, which included training adaptations and manual translations. In addition, efforts to sensitise users about the importance of obtaining generalisable data was crucial to guarantee the quality of the secondary use.

The Swiss landscape is complex with multiple stakeholders, cantonal policies, three languages and varying rhythms. Both top-down and bottom-up movements drive progress. Centralised governance is crucial for dimensions like approval of reimbursement systems, integration into data warehouses, inter-operability of software, definition of national quality indicators, etc. The small-scale pilots have generated greater appetite. Organisations expressed interest in broadening the interRAI portfolio to other populations such as pediatrics, addiction, palliative care, physical disabilities, and informal caregiver needs. To maintain emphasis on clinical utility as a decision support for care planning and quality monitoring, continued efforts are required.

This public health program created synergies between health professionals, researchers, agencies and decision makers on local, federal and international levels. The ambition to bridge these worlds is threefold: increasing acceptability, ensuring coherence and refocusing on the clinical application in order to achieve measurable results. The long-term goal is to move from silos towards full care coordination. Ultimately, quality monitoring and evidence-based policies are envisioned.

As the stepwise rollout of large-scale adoption evolves, the program will continuously need a combination of clinical, academic, and political expertise to innovate the health system in anticipation of changing demographics.

## InterRAI implementation in France

In France, three periods in the development and the implementation of the interRAI instruments over the last twenty years has been identified. The first period was characterised by a research approach regarding improving quality of long-term care. The second one was based on an experimental clinical use into an integrated care model with case management. The third period was carried out by a call for tenders issued by a French national agency. It was decided that interRAI HC was to be implemented for all case managers [44].

At the beginning of 1990, French scientists criticised the health care system and pointed out the major need to improve the definition of functional disability, as well as to improve the collaboration between healthcare and social professionals. The implementation of the InterRAI instruments in 1995 was the opportunity to respond to this critical situation. A study was conducted using the interRAI LTCF in eight nursing homes with two objectives: to identify the acceptability of this instrument by the professionals, and to review the application of interRAI quality indicators in routine care and policy. Moreover, a non-profit organisation was set up in 2000 to promote the development of interRAI tools in France. In the 2000s, some French healthcare teams participated in comparative European studies using interRAI instruments, such as the Ad-HOC study (Aged-Home Care) and the SHELTER study (Services and Health for Elderly in Long Term care [45,46]. In 2009, the French government decided to implement integrated care models for older people living at home. This care model was defined by a collective approach involving all stakeholders in a defined territory addressing the fragmentation of services through interdependent mechanisms and tools. Six components were included, such as areas for cooperation, a shared access-to-services process, the intensive case management for older persons with complex needs, a common instrument for comprehensive assessment, the planning of care services and the information sharing system. The implementation of this integrated care organisation was set up in 17 experimental sites between 2009 and 2012. One of the sites based in the city of Marseille had chosen to implement the interRAI HC for the case managers. After the experimental phase, this integrated care model was implemented all over France and some recommendations were made to implement a new comprehensive instrument. In 2015, a national call for tenders was issued to choose a standardised comprehensive assessment tool for case managers. The criteria for the chosen tool was:

– Existence of a conceptual framework with an international functional classification,
– A multidimensionality approach,
– A relevance to older people with loss of autonomy,
– The possibility to perform the assessment at home,
– The relevance to care planning,
– An overview of resource utilisation,
– Scientific validity,
– The existence of an international network and an active development.

In 2016, the interRAI HC was chosen as the mandatory instrument for the 1,000 case managers starting in France. All sites had the opportunity to use the interRAI CA for the implementation of the shared access-to-services process. A 5-day training was organised for these new professionals to teach them to use the instrument. A dynamic software making the links between the comprehensive assessment, the scales and the CAPs were created to facilitate the learning process.

## InterRAI implementation in Ireland

In 2010 the Older Persons department of Ireland’s Health Service Executive (HSE) established a Working Group to select, pilot and recommend a single assessment tool or suite of tools to be utilised for the assessment of older people nationally in Ireland.

This initiative was aimed at helping to equip the HSE to further key strategic objectives -

– To have the needs of older persons met in the most appropriate setting.
– To provide care that is properly coordinated to support quality and efficiency.
– To maximize value to older persons within the available budget resource.
– To provide demonstrable fairness of access to resources for health and social care, e.g. for Long-Term Residential Care or a Home Care Services.
– To support current national policy on enabling older people to remain at home in independence for as long as possible.

Following a pre-pilot study in 2012 across three sectors, being in Community Care, Long Term Care and Acute care, the Working Group selected the interRAI suite as the most fit-for-purpose for use in Ireland. Government approval was obtained in 2013, and two procurement exercises were completed with vendors appointed to develop the interRAI IT system and associated eLearning component.

InterRAI is being introduced in Ireland on a phased-in bases with initial implementation focusing on older people with more complex needs, therefore, those who may need home care support services, or those in need of long-term care. Subsequent phases will involve the introduction of the interRAI LTCF, interRAI Intellectual Disability (ID), interRAI Carers Needs (CNA), and interRAI PC assessments.

As part of the pre-implementation planning for interRAI, a combined multi stakeholder and multi-disciplinary group was established to adopt interRAI forms and manuals for use in Ireland and these customisations/changes were approved by interRAI.

The first phase of the IT system development commenced in 2014 and a rigorous program of User Acceptance Testing was undertaken. Delays in the software development had incurred due to issues pertaining to offline use of the software system.

In preparation for the national implementation of interRAI, a pilot of the interRAI system commenced in May 2016 across three sites. This pilot involved the training of personnel in these sites who then assessed clients seeking access to long term care or home care supports using the interRAI HC system over an 11-month period. The pilot evaluated the training and education programs, implementation processes, and assessments of data from the three pilot sites.

Following the conclusion of the pilot, national implementation of interRAI within Services for Older People commenced. An implementation framework was established, which details the requirements from both a top down and bottom up approach. National implementation of interRAI Ireland is progressing and is expected to increase in pace in 2022 following the re-procurement of an interRAI IT software vendor.

The pilot, and subsequent roll out of interRAI identified a number of challenges and issues around interRAI implementation:

– The impact of Information and Communication Technology (ICT) – both hardware and software issues
– National policy and ideally supporting legislation is required. Guidelines for these services should incorporate interRAI assessment outputs to inform service urgency, wait lists/prioritisation, appropriate placement, and levels of care
– Training – importance of training for the success of the implementation.
– Regional and local management support is also critical in planning, driving and supporting interRAI implementation.

The resolution of these issues has been of critical importance at both national and regional levels to facilitate interRAI implementation in Ireland.

## InterRAI implementation in Iceland

In the early 90’s, a working committee for interRAI tools in Iceland was established within the Ministry of Health of Iceland. The first steps centered around various pilot projects, but also contained participation in cross-national studies. The version used was MDS 2.0. The initial work led to its implementation nationwide in 1997. The focus was on the financial side, by implementing the 44 group RUG case-mix system. A few years later, quality indicators were developed. Data was being collected during admission to a nursing home, and then subsequently three times per year. Two of these assessments were full assessments and one was a short version, which only served the evaluation of the RUG category. The data was also used for research. Currently, the transformation to the more recent LTCF system is being worked on. A working group from the Medical Directorate of Iceland collects the data and oversees the whole process.

Subsequently, the interRAI HC system has been studied in various small studies and a lengthy process of implementation has been steadily ongoing from 2021 in the capital area of Reykjavik. Initially there were various delays and hurdles to overcome, but the prospect was for an imminent national implementation. It is intended for the information to be used for care-planning and improvement of home care.

The full Mental Health System was translated and computerised for the Department of Psychiatry at the University hospital in Iceland, nevertheless, it has not been put into full use. Currently, it is mostly used for forensic psychiatry and rehabilitation. There were some administrative difficulties relating to changes of administration, which consequently has delayed the process.

The Palliative Care instrument was piloted, however, to date, it has not lead to implementation. The Post-acute care system is being used on one ward within the department of Geriatrics and will soon be adopted on two other units.

The ED screener was implemented at the emergency room at the University Hospital in Iceland with the ED contact assessment used to a lesser degree. The AC instrument was evaluated for implementation into the National University Hospital and was to be used in the preparation of requests for a geriatric consultation. The feasibility of assessing all 75+ people who are admitted to medical and surgical care is currently being evaluated. Research into the Family Caregiver Assessment in Home Care has commenced. Assessment for use in Primary Care is not imminent but will be presented for discussion and evaluation.

The vision is to implement and connect the complete suite of interRAI instruments and let the information flow from one service provider to the next as the client progresses through various parts of the elderly care system in Iceland.

Special attention will be given on how the systematic assessment can transform and improve care of old people in Iceland by informing users and influencing their care pathways. Much more is needed to strengthen implementation and to foster the use of health information. This information is gathered for clinical use and is also used to help both service providers and policy makers in enabling them to make better decisions based on the extensive data the interRAI suite delivers.

## InterRAI implementation in Finland

In 1992, version MDS 1.0 was introduced during the Nordic Gerontology Conference in Denmark, consequently two years later two doctoral theses on the data collected with this instrument was initiated [47,48]. In context of these studies, MDS 1.0 was translated into Finnish and Swedish, which is the two national languages for Finland.

In 2000, the predecessor of the current Finnish Institute for Health and Welfare (THL), known by the name STAKES, launched a 2-year pilot project with voluntary participation of long-term care facilities, in three towns (Helsinki, Kokkola and Porvoo), using the MDS 2.0. During this pilot-project a consortium was formed with the aim of voluntary implementation of the RAI-systems to improve quality of care.

The tasks of the stakeholders of the consortium were:

– Chydenius Institute: to educate nurses how to assess the needs of the residents by using RAI-instruments;
– RAIsoft.ltd: to build up the very first prototype of a software solution to collect data and enhance feasibility of the use of the scales and other output;
– STAKES: to create a benchmarking system in order to compare the case-mix, residents’ needs, and quality of care between the various units; and
– The participating units and or organisations in long term care: to see that every resident was assessed, and the nurses educated to use RAI. The assessments were gathered digitally by STAKES with 6-month intervals, and each participating unit received accordingly a unit, facility, and group level benchmarking report. After the pilot period, it proved to be successful and not a single unit wanted to quit. Home care regions joined the project voluntarily in 2003.

This concept was continued during approximately two decades, with slowly but steadily increasing numbers of participating organisations from both the public and the private sector. In 2018, altogether more than 40% of the residents in residential care, and approximately 35% of the home care recipients were assessed at least twice a year using the instruments. During this time period, the MH, CA, AC, Self-Reported Quality of Life, and ID instruments were piloted. In addition, Finland participated in three European Union financed cross-national MDS or interRAI-based research projects called Ad-HOC, SHELTER and IBenC [31,45,46]. In connection to these studies, the interRAI-LTCF and interRAI HC instruments were translated into the Finnish language.

Based upon discussions with stakeholders, in 2019 the Ministry of Social Affairs and Health was ready to propose to the Finnish Parliament to mandate interRAI-assessment tools. The legislation then changed, and in July 2020, the government launched the Act on Supporting the Functional Capacity of the Older Population and on Social and Health Care Services for Older Persons (980/2012). The goals were to adopt the interRAI instruments in the context of case management and care provision of services for older people in home care and residential care. According to this care act, the deadline for adopting interRAI instruments will be by April 2023. Currently the process of mandating is ongoing.

THL has been assigned, by legislation, to collect the interRAI data nationally, and supervise the availability of instructions, and software (commercially available for organisations). Today, public information as to adopting and using the interRAI instruments, is publicly available at the website (https://thl.fi/en/web/ageing/assessment-of-service-needs-with-the-rai-system).

## InterRAI implementation in Aotearoa, New Zealand

The interRAI instruments are the agreed assessment tools used in Home and Community Support Services (HCSS) for people in Aotearoa New Zealand to support them to stay well in the community and to take increased responsibility for their own health and wellbeing. It is used to determine eligibility for publicly funded HCSS and to enter Aged Residential Care (ARC) where required. A regular assessment is also mandated in ARC to inform the resident’s care plan. Information collected during the assessment is stored in a live data warehouse enabling population data to be collected without any extra effort from the person or their assessor. The aggregated data is available for planning, policy and research purposes to improve population health outcomes.

A review of tools identified interRAI as the best assessment to meet the objectives identified in the New Zealand Best Practice Guidelines “*Assessment Processes for Older People*” [49]. The Guidelines were developed following sector concern over a gap between current and best practice assessment for older people. The vision was to adopt a reliable and meaningful assessment system that could span the range of need and support assessor decision-making. Associated software would promote an objective and transparent assessment process that could be available at every point of care and automatically collect data for evidence-based policy decisions.

In 2004, five District Health Boards (DHBs) successfully piloted the home care assessment as a substitute for the non-validated, subjective assessment in use at the time. Among other things, the pilot evaluation identified that a significant training exercise was required to increase the skill levels of the health professional assessors [50]. In 2007, a business case from DHB Chief Executive Officers led the Ministry of Health (MOH) to allocate sufficient funding to introduce the interRAI Contact, Community Health and Home Care assessments through a national DHB interRAI implementation project (2008–2012).

The project developed an infrastructure to support DHBs to introduce the interRAI assessments within their local model of care. The MOH obtained a license with interRAI to use the integrated suite of assessments and contracted an international software vendor to provide software hosted on a locally built national software platform. This means a single record of a person’s assessment/s is available at their point of care even if the older person’s care provider, care needs or setting changes across the country.

A national training service was established to train and maintain the competency of assessors. Currently there are 10,000 users of the software; 5,000 are health professional assessors who are trained to use one or more assessment versions in their daily work. The education service provides a competency-based curriculum, standardised training materials and national practice standards. Assessors may have assessments selected for quality review and must participate in an annual examination program. Following the recent COVID pandemic all training has moved to a blended model of directed and self-directed learning on-line.

The infrastructure developed during the home care project strongly supported the introduction of the Long-term Care facilities assessment in ARC (2011–2014), and subsequently the use of the Palliative Care instrument across both home and aged residential care.

Key contributing factors in New Zealand:

– A focus on improving health outcomes for each individual.
– An inter-sectoral governance board appointed by the MOH to guide the use of interRAI across Aotearoa New Zealand.
– Strategic engagement of key stakeholders and extensive communication about the value of comprehensive assessment for improving health outcomes.
– A Māori review that approved the items and the process of the assessment. Ongoing work is underway to ensure a culturally appropriate assessment model that meets the needs of Māori.
– A permanent Service funded by MOH for governance, education, software, and data services.

## Dimensions measured in the studies

Table 1 summarises the main aspects of the implementation of the interRAI instruments in the seven countries. The following dimensions were described: the settings where the instruments are being implemented or have been implemented, the territory (nationally or locally), the types of interRAI instruments, a description of the pre-implementation strategy, a description of the implementation design, the methods used for identifying the needs for the instruments, as well as methods for identifying the perceived barriers. The table also shows the main advantages of the interRAI instruments and the main disadvantages or barriers perceived by the users.

**Table 1:** Main aspects of the implementation of the interRAI Suite in seven countries.


ASPECTS OF THE IMPLEMENTATION	BELGIUM	SWITZERLAND	FRANCE	IRELAND	ICELAND	FINLAND	NEW ZEALAND

Settings	Home care and residential care. Rehabilitation and mental health care: under testing.	Home care, residential long-term care, short-stay, day care, assisted living, mental health facilities and acute care	Home care and residential care	Home care and acute care settings	Home care, residential care, acute care, post- acute care and mental health care	Home care, residential care, (MDS pilots in acute care, post- acute care and mental health	Home care, residential care, palliative care and acute care

Territory	First locally in the whole province of Flanders and then nationwide	A mix between nationwide and locally	Locally first and nationwide after	Nationwide	Nationwide (MDS 2.0/LTCF and HC) and at the National University Hospital (AC, PAC, MH)	Nationwide	Nationwide

Types of interRAI Suite instruments to be implemented or already implemented:	Being implemented: interRAI HC, interRAI LTCF, BelRAI Screener, the BelRAI Social Supplement. Under consideration: interRAI PC, interRAI MH, interRAI CMH, supplements of MH, interRAI Rehabilitation Services, Screener for Children and Adolescents	Already implemented: MDS-NH, MDS-HC, interRAI HC and CMH Locally in 1 region: interRAI CU (assessor version), interRAI Screener (Assessment Urgency Algorithm), CPS and ADLH scales integrated in transfer documentation between hospitals and community/residential care To be implemented: Upgrade to interRAI LTCF Under consideration: interRAI Pediatrics and interRAI MH interRAI Check-Up (CU) assessment (self-reported version) interRAI Palliative Care	Already implemented: InterRAI HC for case managers and InterRAI CA for single entry point in integrated model of care To be implemented: interRAI LTCF, PC and AC	Already implemented: interRAI HC and interRAI HC for AC To be implemented: A five-district pilot of the interRAI AC is currently underway in 2022. A pilot of the self- report version of the interRAI CU assessment is in development.	Already implemented: MDS 2.0 to be switched to interRAI LTCF. InterRAI HC partially and increasingly nationwide. Being implemented: ED screener, ED contact Assessment, AC and PAC and MH are all in different stages	Being implemented: HC, LTCF, MDS pilots in acute care, post- acute care, Mental Health Nationwide implementation of the interRAI LTCF and interRAI HC (latest April 2023) Transition from MDS: ongoing	Already implemented: The interRAI Contact, Community Health or interRAI HC,. LTCF, PC in the community or aged residential care (ARC). To be implemented: A five-district pilot of the AC assessment is underway with a national rollout planned for 2022. A pilot of the self-report version of the CU assessment is in development.

Pre-implementation strategy: period and type (top-down, bottom-up, push-pull, etc.)	2006–2016: Feasibility and evaluation studies: firstly top-down, then a bottom-up strategy was put in place.	From 2015: Small scale bottom-up pilots with strong policy support locally	1995–2009: feasibility studies in residential care and nursing home 2009–2015: an experimental integrated care site using the interRAI HC for case management	2012: Pre-Pilot study across Community Care, Long Term Care and Acute care recommending interRAI as most for purpose for implementation in Ireland 2016: National Pilot in 3 locations leading to recommendation for national implementation	1997 to current date: MOH implemented the MDS 2.0 nationwide after some pilot studies in previous five years. The interRAI HC instrument was encouraged by the MOH but not mandated and has taken longer time. The University Hospital assessments are being pushed within different departments.	Pre-implementation strategy MDS voluntary, bottom up from the year 2000 to 2019.	2002: Ministry of Health (MOH) signals the intent to improve assessment systems 2003: an independent Tools Review identified interRAI as best meeting New Zealand’s needs. 2004–2007: Pilot of the interRAI HC in five District Health Boards (DHBs), led by DHBs. 2008–2012: project to implement home and community assessments led by MOH and DHBs 2011–2014: MOH and Aged Care Association pilotted the interRAI LTCF

Implementation design: period and type (top-down, bottom-up, push-pull, etc.)	From 2017 on: Top-down, stepwise implementation	2010–2020: MDS-versions – Top-down with focus on administrative use For nationwide MDS-update: top-down decision and coordination 2020: Push-pull: Larger interest from the field of all sorts of structures and populations hence some applications/conditions demand top-down regulations, development, coordination	From 2017 on: Top down, stepwise implementation of the interRAI HC for the 1,000 case managers in the entire territory	2017: Top down, cross national phased implementation	MDS 2.0 top down and interRAI HC suggested top down. The Hospital systems have been more bottom-up.	When more than one third of all facilities and home care were using MDS, a new legislation about mandatory implementation of the interRAI suite came into force (top-down)	Bottom up initially, then top-down managed implementation in collaboration with relevant stakeholders. 2015: Assessments mandated. Permanent interRAI Service established and funded by MOH includes support for governance, education and support services, software services and data services.

Methods for identifying needs	Feasibility studies, testing of the instruments in real practice, focus groups and intervisions.	Several pilot studies, context analyses, national committees and interregional working groups.	Case studies and focus group with case managers	Pilot study identified challenges which needed to be addressed in order to ensure successful implementation	Needs demonstrated with pilot studies, including cross national studies	Needs of clients, residents, nurses and organisations were collected in pilot studies and in context of cross national studies.	Engagement with key stakeholders. Iterative pilots and projects.

Methods for identifying barriers	SWOT analysis (Strengths, Weaknesses, Opportunities and Threats) and UTAUT analysis (Unified Theory of Acceptance and Use of Technology [51]) during pilot tests	Context analysis Mixed methods evaluations Case studies Structured observations	SWOT analysis	Pilot analysis – SWOT conducted which were categorized into the following categories: ICT, Implementation processes and Training	Not systematically studied and to a variable degree depending on the system	By interviews and discussions with participating nurses and their head nurses, in semi-annual benchmarking meetings. Also by annual board meetings: participating organisations, THL and software companies)	Project management methodology. Strong engagement with key stakeholders. Establishment of a group of highly respected representatives from key government and non- government organisations who had the ability to influence change during implementation.

Main advantages of the interRAI instruments perceived by users	Clinicians and care organisations: Common language across settings., Continuity of care, Improvement and standardization of observations, Involvement of several disciplines in the assessment (multidisciplinary). One single platform to exchange information.	Clinicians and care organisations: Common language across settings Holistic approach: Identifying early decline and domains that are not strictly “medical” Useful in interdisciplinary discussions Facilitates coordination of care by shared information, especially in situations of co-management i.e.	Clinicians and care organisations: Better and comprehensive assessment of resident’s needs Participation of residents and caregivers in their assessment Reinforcement of the multidisciplinary approach Improvement of gerontology knowledge	Clinicians and care organisations: Real time production of decision support information which can be shared by personnel. supports clinical decision-making/corporate decision-making facilitates individualised care planning and integrated care provides case-mix classification at management and systems level	Clinicians and care organisations: Common language across settings, Resource Utilisation Quality indicators Research	Clinicians and care organisations: Makes the demands of the work in older people’s care visible Helps to identify clients’ needs (how and what to observe) and create the individual care plan Helps to monitor client level outcomes Shows the quality of the performed work Helps the workers to discuss with relatives, and informal carers	Clinicians and care organisations: Common language across settings Decision support properties for assessors to inform care Transparent and objective review of assessment quality Supports prioritisation and eligibility processes One single platform (assessment record) to exchange information.
	Policy makers: Benchmarking across organisations. Resource planning. Evidence based decision making	multiple agencies care for the same person simultaneously (e.g. home care and day care service) policy makers: Decision support based on valid and comparable data Potential to support and monitor public health programs across the care continuum (quality, care trajectories, care expenses, alternative care structures, community-enhancing initiatives, comparisons) Interoperability for electronic health records and integration in data warehouse Focus from acute problems to prevention and identifying early decline The 2-step use of interRAI CU and the interRAI HC to differentiate length of assessment according to the care complexity is more efficient The change to broaden the assessors’ job to non-nursing disciplines is greatly appreciated (e.g. social workers, occupational therapist, nutritionist) Difficult to change existing practice and to change routine processes	having a unique assessment with series of applications quality indicators produced by the assessment For case managers using the interRAI HC: making the link between the comprehensive assessment and the care planning Legitimation of their role as case managers Police makers and care managers were not involved in the evaluation	enables quality monitoring, benchmarking, and service improvement informs eligibility criteria for access to services supports prioritisation of services access based on assessed need targets priority groups or identifies groups that are at relative risk of adverse outcomes Policy Makers: Enhances knowledge of client care populations Identifies service need/service improvements Informs allocation of resources and prioritisation of services based on real-time assessed needs Can determine eligibility for services Prioritises service users who are most in need Provision of data for performance monitoring and quality assurance Allows managers to track and compare their organisations’ responses to quality of care issues Better management of services/resources Demonstration of effective care/value for money		Nursing leaders: Helps in dividing tasks between distinct professions, in units Shows gaps in staff knowledge/strengths in expertise and helps planning of further education of the existing staff, and recruiting new type workers Organisational leaders: tool for regulative procedures setting strategic/tactical goals for the organisation protects from false accusations (from media, individuals) Regional leaders case-mix and quality comparisons of public and outsourced care providers Though benchmarking follow-up of performed policies issues of integrated care can be solved in the future Payment systems piloted National leaders/Policy makers: Creating new policies and follow-up of the outcomes (safety and quality of care, gate-keeping criteria, staffing ratio etc.) Identifying need for new legislations/guidelines	Policy makers and researchers: Collection of information once in a live national database that can be used for many purposes, such as understanding the needs of the assessed population, making evidence based decisions, understanding of health outcomes (equity), allowing case mix funding and resource planning

Main disadvantages or barriers perceived by users	Electronic records and IT infrastructure is not always available. Overlap with other instruments already used in the care practice. Low involvement of GPs. Time consuming at first but time saved after first assessment is filled out. Fear that clinical judgement will become redundant.	Difficult to learn Needs specific coaching to integrate it into team processes on the field Overlap with existing assessments in use Fears of administrative charge (MDS-NH was purely used for funding) Software is often non-attractive as some vendors do not use the full potential and are not focused on user-friendliness Low involvement of GPs The locally-made derived versions in the Swiss context are different from the original internationally validated system and therefore confusing (Swiss version of RUGs, QIs, item content, development of RAI-like instruments…) Data literacy is often low Quality of trainings varies Technical software support for longitudinal data visualisations across settings is not developed yet in Switzerland No integration in national electronic health record yet For inter-institutional use, guidelines and communication structures are needed	Fears of the standardisation practices Identification of work-organisation problems in nursing home Lack of connection between research purpose and a routine clinical utilization Lack of appropriation of other applications	Length of assessment – time consuming ICT connectivity/offline use Software/hardware issues	Perceived to be time consuming tardy integration into the Electronic Medical Record and the Nurses Records limited support to utilise the information to change practice.	Need for education in how to assess the client needs interRAI-tools assessment process is time consuming bedside education of new workers is time consuming need for education how to lead and manage with help of interRAI-tools cautiousness among staff and leaders about how to use the tools (human makes the decisions) incompatibility or only partial compatibility of interRAI systems with patient/client records within jurisdictions incompatible software solutions have been a barrier for integrated care between jurisdictions	Conceptual and terminology challenges for assessors changing from a subjective and narrative questionnaire type of assessment to a software supported and transparent assessment system with decision support properties. Trust and confidence in a new assessment process. Potential loss of clinical autonomy. Trust and confidence in the software system. Interoperability barriers if the software does not connect with other systems in use. Trust and confidence in the data. Resources or knowledge to analise or use the data. Time consuming during the learning period.


Table 2 describes the main steps taken towards the implementation of the interRAI instruments in the seven countries.

**Table 2:** Main steps taken towards implementation of the interRAI instruments in the seven countries.


IMPLEMENTATION STEPS	BELGIUM	SWITZERLAND	FRANCE	IRELAND	ICELAND	FINLAND	NEW ZEALAND

1	Translation into national languages (Dutch, French and German). Adaptation of the instruments and manuals to the Belgian social care and health care settings.	Evaluation of the existing use of assessment instruments in general and interRAI in particular. Exploration of the context (clinical, political). Mapping of the stakeholders (roles – agendas).	Translation into the French language.	Selection of interRAI- system of Assessments and Customisation/locatisation of instruments, associated manuals, and CAPs for use in the Irish context.	Translation into Icelandic	Translation into Finnish and Swedish languages	Transparent process to select interRAI (Tools Review). Business cases approved Project management infrastructure established. Active communication with the sector.

2	Comparison between the instruments to make them compatible across settings.	Developing a roadmap to introduce the newest generation interRAI Suite with focus on clinical use. Organisation of a conference to introduce the public health program to the field.	Participation in comparative studies with interRAI LTCF and HC instruments	Development of IT platform (Testing/Training and live applications) and associated eLearning System	Establishment of interRAI Iceland committee which worked within the MOH and later within the Medical Director General.	Back translation of the Finnish version. A version of Swedish translation received from Sweden, and modified according the language used in Finland.	Acceptance from Māori for the items and assessment process. Customisation of demographic items and New Zealand English terms.

3	Creation of an IT platform allowing professional caregivers from several disciplines to fill out the instruments in a multidisciplinary way (physicians, nurses, physiotherapists, occupational therapists, social assistants, etc.).	Creation of a legal framework (a decree) enhancing electronic health records and a common language between health settings to reinforce coordination and continuity of care. Strong political signal for policy support.	Creation of a non-profit organisation InterRAI France to promote the implementation.	Development of Training Programme tailored to user groups (Assessors, Decision Makers, etc.)	IT platform developed in Icelandic and compatible for all assessment forms.	Creation of Educational model how to assess the needs, and understand the scales (Chydenius Institute) Building up a commercial feasible software for data collection and creation of individual care plan (RAIsoft.ltd) Creation of first version of benchmarking the outcomes of care including: 1) safe digital transference of the person level data (STAKES/THL) Creation of unit/organization organisation/national level feed-back reports Creation of different types of seminars according the needs of participants	Single software platform developed. National training service developed. Contract with international software vendor. Contract with international e-learning system vendor.

4	Adaptation of some CAPs to the Belgian care setting, with evidence-based guidelines.	Defining, designing small-scale pilots. Guaranteeing the conditions: software, training, local support, project managers, budget, academic participation.	Implementation of integrated care model with intensive case management	Programme of User Acceptance Testing and Live Testing of IT system and associated processes/workflows.	Small supportive group for training and supervision from within the Medical Directorate of Iceland.	Collecting data of unit-level costs of care. Due to scattered way of governance, it turned out impossible to find or calculate information on costs, in compatible ways. In 2000 there were more than 400 independent regional jurisdictions, with non-compatible ways of calculating costs, in the country, and in 2022 still over 300.	A (4 year) – national implementation project that encouraged ownership and autonomy at the local level as long as national project parameters were met. Change management techniques for clinicians and managers. Data services strategy developed. Demonstrations of the aggregated data and potential use to inform planning.

5	Creation of an online manual for easy access while filling out the instruments – the BelRAI wiki website.	Translation and adaptations to local context of multiple instruments with special attention to coherence between languages and between instruments.	A top-down decision by the French national agency the “Caisse Nationale Solidarité Autonomie” (CNSA) to implement the interRAI HC for the case managers.	11-month pilot in 3 sites nationally	Caregiver groups and organisations have established working groups around implementation and to work with quality indicators.	Improving benchmarking reports Improving education Training experts -e-learning Improving contents of the seminars	Strong sector engagement through meetings, conferences and successful pilots and projects. Focus on the value of interRAI assessment for the person’s role, their client’s welfare and their organisational efficiency.

6	Organisation of training for professional caregivers and train-the-trainer to diffuse the knowledge in a more rapidly way.	Multiple pilots of various interRAI instruments in diverse populations, diverse settings, with varying length in time and size.	Development of a training program based on a dynamic software, making the links between the comprehensive assessment, the CAPS and the scales	Development of implementation framework for roll out of interRAI implementation in CHO regions.	Implementation evaluated by KPMG (MDS 2.0 and HC)	Gaining mandatory implementation	interRAI New Zealand established with ongoing funding from Ministry of Health for: Governance Board, Education and Support Service, Software Service, Data Services, Website and newsletters

7	Development of e-learning.	Regular free-of-charge conferences on evidence-based care and various forms of interRAI applications locally and internationally. Create a community of users and future users. Preparing upscaling from pilot to integration in routine use: train-the-trainer, e-learnings, funding, licensing, support	Training the interRAI trainers of the case managers	Development of IT equipping and support processes in conjunction with National HSE IT department.			Establishment of an iterative process for introducing new interRAI assessment versions building on sector support for example introducing the Palliative Care assessment to the sector Building relationships with researchers and developing an interRAI research community

8	Designing several pilot projects.	Policy considerations. Preparation and rollout of the switch from MDS-version to Suite. Train-the-trainer, licensing software vendors, updates manuals, progress evaluation.		Re-procurement exercise for alternative software provider following conclusion of initial contract.			Undertaking and responding to independent reviews Implementation requirements (2005) The LTCF Implementation (2018) Review of Service Design (2020)


## Discussion

This paper summarised the process of implementation of the interRAI instruments in seven countries. The phases related to the period of testing and evaluation of the use of the instruments were described, as well as the dimensions of the implementation process and the steps taken towards implementation to identify best practices.

The results showed that all countries first started with a stepwise strategy, often a bottom-up approach that was however initiated by government initiatives, consisting of pilot studies to evaluate the use of the instruments in care practice. Later on, after the instruments showed their added-value, a top-down approach was taken towards implementation.

Some main advantages of the implementation of the interRAI instruments are linked to the aspect of continuity and integration of care. A common language across settings, the involvement of several disciplines in the assessment (multidisciplinary) and the development of IT platforms to exchange information across professionals, organisations and settings were cited as essential tools to achieve care integration and continuity of care. These advantages are in congruence with the aims of the OECD Framework and Scorecard for People-Centered Health. The OECD reports that countries have recently leveraged digital tools to improve integration, but, despite the progress in the uptake of electronic health records, establishing linkages and integration between systems has been slow, with primary care often excluded from close electronic integration with other settings. Fewer than 40% of the OECD countries reported to regularly conduct linkage projects with primary care data. In this framework, primary care serves as an important coordinating node for care management – particularly for people with chronic conditions, ensuring a strong continuity of care, as clients transition between settings of the health care systems. The framework ensures the need for the integration of health systems, as well as the importance of the systematic measurement of clients’ experience and outcomes, to foster the use of quality measurements (indicators), as well as to allow international comparability between countries [1].

In addition, as the interRAI instruments are comprehensive, they offer a holistic approach to the care practice, enabling care professionals to identify early decline and domains that are not strictly “medical”, which is useful for interdisciplinary discussions. In practice, this brings a shift of the focus from acute problems to prevention and enables care professionals to first identify and prevent early decline. In addition, the standardised functional data help clinicians to objectivise clinical changes over time. Another shift mentioned was the broadening of the assessors’ job from strictly clinical disciplines to non-nursing disciplines, which seems to be greatly appreciated in practice.

Another added-value of the interRAI Suite is that it works as a decision support for care planning, based on valid and standardised data. In France, in particular, the implementation of the interRAI assessment brought more legitimation to the role of the case manager, who found the link between the comprehensive assessment and the care planning very useful. A recent study identified the most relevant barriers and facilitators for the use of a clinical decision support system (CDSS) reported by 581 physicians from 11 European countries. Technical issues, having to indicate a reason for overriding an alert and unclear advice were perceived as the most important barriers, as well as time-expenditure of the CDSS, the need of integration with existing systems and accurate advice for medically complex clients. Relevant facilitators were a CDSS that is beneficial to patient care, easy-to-use and easily accessible, contributed to increased work efficiency, fits into the physician’s workflow and supportive to the decision-making process [52].

Most countries reported technical aspects as barriers for implementation, such as difficulties to access electronic records and unavailability of IT infrastructure, as well as a low interoperability when the interRAI software is not compatible with other systems already in use. These compatibility issues make the assessment very time consuming, as the instruments may overlap with other data collected via other software used by the organisations.

A recent systematic review showed that electronic health assessments can inform healthcare policy making and achieve evidence-based health care. Assessments should be standardized and should have a stepped-approach tailored to the functional characteristics of the eHealth services. The review reported that these assessments can improve transparency, comparability, and efficiency, as well as facilitate collaboration across healthcare systems in decision and policy making in digital health care [53]. Another systematic review identified an increase in productivity/efficiency and quality of data or care, as well as better data management. The top three barriers were missing data, no standards for interoperability, and a time consumption for training of users [54]. Our results are in line with these studies.

Training in the use of the instruments was mentioned as an important step towards implementation. According to users, as long as the quality of the training is poor, problems in the quality of the data will remain. Moreover, the low involvement of the general practitioner in the assessment is perceived as a barrier, especially in the home care setting, as they are usually the first to identify complex situations. Many countries also reported a fear in the standardisation of practices and the potential loss of clinical autonomy. Enhancing trust in the software and in the data can improve assessment involvement and the multidisciplinary use of the information in the care planning.

At the management level, the advantages mentioned related to the possibilities for benchmarking across organisations, sectors and even countries, resource planning and evidence-based decision making. In addition, the assessments provide information which can be used for allocation of resources and prioritisation of services, identifying service users who are most in need of care. By providing data for performance monitoring and quality assurance, managers are able to track and compare their organisations responses to quality of care issues. This makes it possible to demonstrate service effectiveness, which generates value for money. Putting this into place however is burdensome and needs specific competences.

For policy makers, the possibilities to support and monitor public health programs across the care continuum show great potential, as well as the possibility of benchmarking across countries. In addition, the interoperability for electronic health records and integration in data warehouses makes it possible to use the data for evidence-based policy making. The use of the assessments enables quality monitoring and can inform decisions about eligibility criteria for access to services. This supports prioritisation of services based on identified needs, targeting priority groups or groups that are at higher relative risk of adverse outcomes [55,56,57,58,59,60,61]. These measures can help to create equity and a more targeted financing for the care.

Governments are keen to foster integrated care but the conditions for implementation are not always favorable [62]. It is important to inform policy makers and organisations on how to organise integrated care and how to use available evidence to guide professionals into a higher degree of collaboration [63]. In addition, it is not always clear to professionals how to use evidence-based outcomes to create care plans in a multidisciplinary way or how to use these outcomes to match services to clients. The interRAI outcomes make it possible to create care plans effectively and to prepare the multidisciplinary meetings, so that each caregiver, together with the client and the care provider, can decide which are the important problems and needs to address [9]. Collaboration involving all stakeholders can therefore happen in a structured way supported by the assessment and the IT platform. In addition, with new interRAI assessments also involving the dimension of informal care, the whole care situation (formal and informal) can be evaluated and addressed [64,65,66].

## Lessons learned

Research clearly shows that the interRAI suite of instruments has a great potential to:

– Enhance multi and interdisciplinarity,
– Allow the transfer of client-centered data between and within different care settings,
– Enhance integrated care,
– Improve quality of care.

In all seven countries, an IT platform was developed to support the assessment and its features, as well as to allow information sharing. However, the development and implementation of information technology is a gradual and long process, which also has to count on the trust and acceptability of caregivers to work with the new IT platform. Users must be convinced that the platform is safe and that the electronic assessments can contribute to quality of care and to benefits in the care practice.

## Conclusion

In reality, the use of evidence is a process that requires a cultural shift from caregivers at all levels of health and social care delivery. Information about the implementation process of the interRAI Suite in different countries can help investigators and policy makers to better plan the implementation process of these health assessment tools. This research has shed light on the advantages and pitfalls of using the interRAI Suite of instruments and proposes some approaches to overcome difficulties. Pointing out the benefits of the system can motivate users and improve adoption in other countries. Adequate staffing, appropriate information technology, professional collaboration and continuous training can contribute to the implementation of the interRAI instruments in the care practice.

## Abbreviations

ADLH – interRAI Activities of Daily Living Hierarchy scale

ARC – Aged Residential Care

BelRAI Screener – Belgian Screener for Home Care

BelRAI Social Supplement – Belgian Social assessment

CAPs – Clinical Assessment Protocols or Collaborative Action Points

CDSS – Clinical Decision Support System

CMH – Community Mental Health instrument

CPS – interRAI Cognitive Performance Scale

DHBs – District Health Boards

HCSS – Home and Community Support Services

HSE – Health Service Executive

ICT – Information and Communication Technology

interRAI AC – interRAI Acute Care instrument

interRAI CA – interRAI Contact Assessment instrument

interRAI CNA – interRAI Carers Needs Assessment

interRAI CMH – interRAI Community Mental Health instrument

interRAI CU – interRAI Check-up assessment

interRAI ED Screener – interRAI Emergency Department Screener instrument

interRAI HC – interRAI Home Care instrument

interRAI ID – interRAI Intellectual Disability

interRAI LTCF – interRAI Long-Term Care Facilities instrument

interRAI MH – interRAI Mental Health instrument

interRAI PC – interRAI Palliative Care instrument

interRAI Self-Reported QOL – interRAI Self-Reported Quality of Life

LTCF – Long Term Care Facility

MDS-NH – Minimum Data Set for Nursing Homes

MDS-HC – Minimum Data Set for Home Care

MH – Mental Health Instrument

MOH – Ministry of Health

OECD – The Organisation for Economic Co-operation and Development

RUG – Resource Utilization Group

SWOT Strengths, Weaknesses, Opportunities, and Threats analysis

THL – The Finnish Institute for Health and Welfare

USA – The United States of America

## References

[1] OECD. Health for the People, by the People: Building People-centred Health Systems. OECD Health Policy Studies. Paris: OECD Publishing; 2021. DOI: 10.1787/c259e79a-en

[2] Goodwin N. Understanding Integrated Care. International Journal of Integrated Care. 2016; 16(4): 6. DOI: 10.5334/ijic.2530 PMC5354214 28316546

[3] Carpenter GI, Hirdes JP. Using interRAI assessment systems to measure and maintain quality of long-term care in OECD/European Commission: A Good Life in Old Age? Monitoring and Improving Quality in Long-term Care, OECD Health Policy Studies. OECD Publishing, 2013; 93–139. DOI: 10.1787/9789264194564-7-en

[4] Fries B, Fahey C. Implementing the resident assessment instrument: case studies of policymaking for long-term care in eight countries. New York: Milbank Memorial Fund; 2003.

[5] Morris J, Hawes C, Fries BE, Phillips C, Mor V, Katz S. Designing the National Resident Assessment Instrument for Nursing Homes. The Gerontologist. 1990; 30(3): 293–307. DOI: 10.1093/geront/30.3.293 2354790

[6] Hirdes JP, Ljunggren G, Morris JN, et al. Reliability of the interRAI suite of assessment instruments: a 12-country study of an integrated health information system. BMC Health Serv Res. 2008; 8: 277. DOI: 10.1186/1472-6963-8-277 19115991 PMC2631461

[7] Bernabei R, Landi F, Gambassi G, Sgadari A, Zuccala G, Mor V, Rubenstein L, Carbonin PU. Randomized trial of impact of model of integrated care and case management for older people living in the community. BMJ. 1998; 316(7141): 1348–1351. DOI: 10.1136/bmj.316.7141.1348 9563983 PMC28532

[8] Morris J, Carpenter I, Berg K, Jones RN. Outcome measures for use with home care clients. Can J Aging. 2000; 19(2): 87–105. DOI: 10.1017/S071498080001391X

[9] Vanneste D, Declercq A. The development of BelRAI, a web application for sharing assessment data on frail older people in home care, nursing homes and hospitals. In: Muller S, Kubitschke L (eds.), Beyond Silos – The why and how of integrated eCare. 2014; 202–226 Hershey IGI. ISBN: 9781466661387. DOI: 10.4018/978-1-4666-6138-7.ch010

[10] Fries BE, Schneider DP. Foley WJ., Gavazzi M, Burke R, and Cornelius E. Refining a Case-mix Measure for Nursing Homes: Resource Utilization Groups (RUG-III). Medical Care. 1994; 32: 668–685. DOI: 10.1097/00005650-199407000-00002 8028403

[11] Dellefield ME. Using the resource utilization groups (RUG-III) system as a staffing tool in nursing homes. Geriatr Nurs. 2006; 27: 160–5. DOI: 10.1016/j.gerinurse.2006.02.004 16757387

[12] Bjorkgren MA, Fries BE, Shugarman LR. A RUG-III case-mix system for home care. Can J Aging. 2000; 19(Suppl 2): 106–125. DOI: 10.1017/S0714980800013921

[13] De Almeida Mello J, Declercq A. (contr.) Towards continuity of care and evidence-based decision making: The implementation of the interRAI Suite in Belgium. Presented at the World interRAI Conference; 2020. Leuven, 03 Feb 2020–05 Feb 2020.

[14] Wellens N, Monod S, Grandchamp C, Audard M, Matos Queiros A. A Swiss Health Policy Programme building continuity of care by bridging clinicians, researchers and decision-makers. Presented at the World interRAI Conference. Leuven; 03 Feb 2020–05 Feb 2020.

[15] Vereker N, Regan IO. A review of the implementation of interRAI in Ireland. Presented at the World interRAI Conference. Leuven; 03 Feb 2020–05 Feb 2020.

[16] de Stampa M, Cerase V, Bagaragaza E, Lys E, Alitta Q, Gammelin C, Henrard J-C. Implementation of a Standardized Comprehensive Assessment Tool in France: A Case Using the InterRAI Instruments. Presented at the World interRAI Conference. Leuven; 03 Feb 2020–05 Feb 2020. 10.5334/ijic.3297 PMC6095084 30127689

[17] Meehan B. Where Angels Fear to Tread: New Zealand’s implementation of the interRAI suite of instruments. Presented at the World interRAI Conference. Leuven; 03 Feb 2020–05 Feb 2020.

[18] Jónsson P. interRAI implementation in Iceland. Presented at the World interRAI Conference. Leuven; 03 Feb 2020–05 Feb 2020.

[19] Yin RK. Case study research: Design and methods 2003. (3rd ed.). Thousand Oaks, CA: Sage.

[20] Creswell JW. Qualitative inquiry and research design: Choosing among five approaches (3rd ed). 2013; Thousand Oaks, CA: Sage

[21] Denzin NK, Lincoln YS. (Eds.) The SAGE handbook of qualitative research (4th ed.). 2011b; Thousand Oaks, CA: Sage.

[22] Fernandez Maria E, ten Hoor Gill A, van Lieshout S, Rodriguez Serena A, Beidas Rinad S, Parcel Guy, Ruiter Robert AC, Markham Christine M, Kok G. Implementation Mapping: Using Intervention Mapping to Develop Implementation Strategies. Frontiers in Public Health. 2019; 7. DOI: 10.3389/fpubh.2019.00158 31275915 PMC6592155

[23] Baxter P, Jack S. Qualitative Case Study Methodology: Study Design and Implementation for Novice Researchers. The Qualitative Report. 2008; 13(4): 544–559. DOI: 10.46743/2160-3715/2008.1573

[24] De Lepeleire J, Falez F, Swin C, Ylieff, M, Pepersack T, Buntinx F. INTERFACE rapport; 2005. Brussel.

[25] Declercq A, Flamaing J, Gosset C, Milisen K, Moons P, Collard J, Delye S, et al. BelRAI VI – Wetenschappelijk onderzoek met betrekking tot de pilootprojecten voor het transmuraal gebruik van het BelRAI-instrument; 2011. Brussel.

[26] Declercq A, Gosset C, Paepen B, de Almeida Mello J, Vanneste D, Detroyer E, Milisen K, et al. Actieproject BelRAI II: Haalbaarheid van de RAI-methode in België. Brussel; 2008.

[27] Declercq A, Gosset C, Paepen B, de Almeida Mello J, Vanneste D, Detroyer E, Spruytte N, et al. Actieproject BelRAI III: Haalbaarheid van de RAI-methode in België; 2009. Brussel.

[28] Declercq A, Gosset C, Wellens N, Collard J, Filee D, Londot A, Polome L, et al. Actie-onderzoek naar het gebruik van het RAI-instrument in de geriatrische dagziekenhuizen, de rust- en verzorgingstehuizen, de dagcentra en de geïntegreerde diensten voor de thuiszorg; 2007. Brussel.

[29] Wellens N, Deschodt M, Boonen S, Flamaing J, Gray L, Moons P, Milisen K. Validity of the InterRAI Acute Care Based on Test Content: A Multi-center Study. Aging Clinical and Experimental Research. 2011; 23(5): 476–86. DOI: 10.1007/BF03325244 22526080

[30] de Almeida Mello J, Van Durme T, Macq J, Declercq A. Interventions to delay institutionalization of frail older persons: design of a longitudinal study in the home care setting. BMC PUBLIC HEALTH, 2012; 12. Art.No. ARTN 615. DOI: 10.1186/1471-2458-12-615 PMC3490799 22867442

[31] van der Roest HG, van Eenoo L, van Lier LI, et al. Development of a novel benchmark method to identify and characterize best practices in home care across six European countries: design, baseline, and rationale of the IBenC project. BMC Health Serv Res. 2019; 19: 310. DOI: 10.1186/s12913-019-4109-y 31092244 PMC6521361

[32] Hermans K, Spruytte N, Cohen J, Van Audenhove C, Declercq A. Informed palliative care in nursing homes through the interRAI palliative care instrument: a study protocol based on the medical research council framework. BMC Geriatrics. 2014; Art.No. DOI: 10.1186/1471-2318-14-132 PMC4280705 25479633

[33] Vermeulen B, Van Eenoo, L, Vanneste, D, Declercq A. Naar een getrapt gebruik van BelRAI met de BelRAI Screener. 2015; Leuven: LUCAS.

[34] Van Doren S, Hermans K, Declercq A. Towards a standardized approach of assessing social context of persons receiving home care in Flanders, Belgium: the development and test of a social supplement to the interRAI instruments. BMC HEALTH SERVICES RESEARCH. 2021; 21(1): Art.No. ARTN 487. DOI: 10.1186/s12913-021-06453-w PMC8140469 34022861

[35] Programme coRAI: Renforcer la continuité et la coordination des soins par un langage commun (interRAI©). Website accessed on March 2022: https://www.vd.ch/toutes-les-autorites/departements/departement-de-la-sante-et-de-laction-sociale-dsas/direction-generale-de-la-sante-dgs/projets/news/10804i-renforcer-la-continuite-et-la-coordination-des-soins-par-un-langage-commun/.

[36] DÉCRET 800.033 sur le développement d’outils et de processus favorisant la continuité et la coordination des soins (DCCS), 7 december 2016, Le Grand conseil du Canton Vaud, Lausanne, Switzerland. Published on website: https://www.lexfind.ch/fe/fr/tol/24359/fr.

[37] Cattagni Kleiner A, Debons J, Pin S, Simonson T, Santos-Eggimann B, Seematter-Bagnoud L. Implémentation clinique de la démarche interRAI dans les EMS vaudois: évaluation d’un projet pilote. 2019; Lausanne, ISSN: 1660-7104. DOI: 10.16908/issn.1660-7104/297

[38] Seematter-Bagnoud L, Cattagni Kleiner A, Fustinoni S, Santos-Eggimann B. Implémentation d’une démarche interRAI d’évaluation des clients des CMS vaudois: bilan d’un projet pilote; 2019; Lausanne, ISSN 1660-7140. DOI: 10.16908/issn.1660-7104/298

[39] D’Onofrio A, Chenevey AH, Wellens NIH. InterRAI et coordination dans les SAMS: Évaluation du projet clinique; 2021. Lausanne, Switzerland.

[40] Morris J, Howard E, Geffen L, Hirdes J, Hogeveen S, Berg K, et al. interRAI Check Up© version 10.1. Instrument d’évaluation et manuel. Version adaptée pour utilisation uniquement dans le cadre du programme coRAI de l’Etat de Vaud, Suisse avec la contribution de NIH Wellens, M Mayer de la Direction Générale de la Santé Vaud et A Dulimbert, M Karlen, N Bacq de l’AVASAD, 2019; Lausanne, Switzerland.

[41] Costa PA. interRAI Assessment Urgency Algorithm© (AUA) – interRAI Screener version 9.3.0. Guide d’évaluation et manuel d’utilisation pour l’évaluation de dépistage. Version adaptée pour utilisation uniquement dans le cadre du programme coRAI de l’Etat de Vaud, Suisse avec la contribution de NIH Wellens, M Mayer de la Direction Générale de la Santé Vaud et A Dulimbert, M Karlen, N Bacq de l’AVASAD, 2019; Lausanne, Switzerland.

[42] Wellens NIH, Barman PO, Oettli A. Trajectoires de soins: apparier des données cliniques et démographiques pour illustrer des parcours dans le système de santé, Statistique Vaud & Direction générale de la santé, 2022; Lausanne, Suisse.

[43] Mulla R, Turcotte LA, Wellens NIH, Angevaare MJ, Hébert PC, Heckman G, Weir J, van Hout H, Hirdes JP. Prevalence and predictors of influenza vaccination in long-term care homes: A cross-national retrospective observational study. BMJ Open. 2022; 12: e057517. DOI: 10.1136/bmjopen-2021-057517 PMC9016404 35437252

[44] de Stampa M, Cerase V, Bagaragaza E, Lys E, Alitta Q, Gammelin C, Henrard JC. Implementation of a Standardized Comprehensive Assessment Tool in France: A Case Using the InterRAI Instruments. Int J Integr Care. 2018 Apr 18; 18(2): 5. PMID: 30127689; PMCID: PMC6095084. DOI: 10.5334/ijic.3297 PMC6095084 30127689

[45] Carpenter I, Gambassi G, Topinkova E, Schroll M, Finne-Soveri H, Henrard JC, Garms-Homolova V, Jonsson P, Frijters D, Ljunggren G, Sørbye LW, Wagner C, Onder G, Pedone C, Bernabei R. Community care in Europe. The Aged in Home Care project (AdHOC). Aging Clinical Experimental Research. 2004 Aug; 16(4): 259-69. DOI: 10.1007/BF03324550 15575119

[46] Onder G, Carpenter GI, Finne-Soveri H, Gindin J, Frijters D, Henrard JC, Nikolaus T, Topinkova E, Tosato M, Liperoti R, Landi F, Bernabei R. SHELTER project. Assessment of nursing home residents in Europe: the Services and Health for Elderly in Long TERm care (SHELTER) study. BMC Health Services Research. 2012; 12(1): 5. DOI: 10.1186/1472-6963-12-5 22230771 PMC3286368

[47] Finne-Soveri Harriet. Daily pain in institutional long-term care. Doctoral thesis, 2001; STAKES

[48] Björkgren M. Case-Mix Classification and Efficiency Measurement on long-term Care of the Elderly. Doctoral thesis; 2002. STAKES.

[49] Martin JO, Martin IR. Assessment of community dwelling older people in New Zealand: a review of comprehensive and overview assessment tools. Available at: www.nzgg.org.nz/guidelines/0030/Final_Report_tools_review.pdf.

[50] Weidenbohm K, Parsons M, Dixon R, Keeling S, Senior H, Brandt T. The interRAI evaluation: The Exploration of the interRAI training program implemented across five District Health Boards across New Zealand; 2008; New Zealand: Ministry of Health.

[51] Vanneste D, Vermeulen B, Declercq A. Healthcare professionals’ acceptance of BelRAI, a web-based system enabling person-centred recording and data sharing across care settings with interRAI instruments: a UTAUT analysis. BMC Med Inform Decis Mak. 2013; 13: 129. DOI: 10.1186/1472-6947-13-129 24279650 PMC4222843

[52] Ploegmakers KJ, Medlock S, Linn AJ, et al. Barriers and facilitators in using a Clinical Decision Support System for fall risk management for older people: a European survey. Eur Geriatr Med; 2022. DOI: 10.1007/s41999-021-00599-w 35032323

[53] Vis C, Bührmann L, Riper H, Ossebaard HC. Health technology assessment frameworks for eHealth: A systematic review. International Journal of Technology Assessment in Health Care. 2020; 1–13. DOI: 10.1017/S026646232000015X 32297588

[54] Kruse CS, Stein A, Thomas H, Kaur H. The use of Electronic Health Records to Support Population Health: A Systematic Review of the Literature. J Med Syst. 2018 Sep 29; 42(11): 214. DOI: 10.1007/s10916-018-1075-6 30269237 PMC6182727

[55] Foebel A, Ballokova A, Wellens NIH, Fialova D, Milisen K, Liperoti R, Hirdes J. A retrospective, longitudinal study of factors associated with new antipsychotic medication use among recently admitted long-term care residents. BMC Geriatrics. 2015; 15(1). art.nr. 128. DOI: 10.1186/s12877-015-0127-8 PMC4615888 26482028

[56] McArthur C, Saari ME, Heckman GA, Wellens NIH, Weir J, Herbert P, Turcotte L, Jbilou J, Hirdes JP. Evaluating the effect of COVID-19 pandemic lockdown on long-term care residents’ mental health: a data-driven approach in New Brunswick. Journal of the American Medical Director’s Association; 2020. DOI: 10.1016/j.jamda.2020.10.028 PMC7587131 33232682

[57] Mulla R, Turcotte LA, Hébert PC, Heckman G, Weir J, Wellens NIH, Angevaare MJ, van Hout H, Hirdes JP. Prevalence and predictors of influenza vaccination in long-term care homes: A cross-national retrospective observational study; 2021 Submitted. DOI: 10.1136/bmjopen-2021-057517 PMC9016404 35437252

[58] Klunder JH, Bordonis V, Heymans MW, van der Roest HG, Declercq A, Smit JH, Garms-Homolova V, Jonsson PV, Finne-Soveri H, Onder G, Joling KJ, Maarsingh OR, van Hout HPJ. Predicting unplanned hospital visits in older home care recipients: a cross-country external validation study. BMC GERIATRICS. 2021; 21(1), Art.No. ARTN 551. DOI: 10.1186/s12877-021-02521-2 PMC8515741 34649526

[59] Heckman GA, Hirdes JP, Hébert P, Costa A, Onder G, Declercq A, Nova A, Chen J, McKelvie RS. Assessments of Heart Failure and Frailty-Related Health Instability Provide Complementary and Useful Information for Home-Care Planning and Prognosis. Can J Cardiol. 2021; 37(11): 1767–1774. DOI: 10.1016/j.cjca.2021.07.009 34303783

[60] Garms-Holová V, Declercq A, Finne-Soveri H, Notthoff N, van der Roest HG, van Hout H. Adverse Life Events: Do Home Care Clients Have Resources for Mastering them? Frontiers in Medicine. 2021; (8). Art.No. 522410, 1–9. DOI: 10.3389/fmed.2021.522410 PMC7973033 33748153

[61] Hirdes JP, van Everdingen C, Ferris J, Franco-Martin M, Fries BE, Heikkilä J, Hirdes A, Hoffman R, James ML, Martin L, Perlman CM, Rabinowitz T, Stewart SL, Van Audenhove C. The interRAI Suite of Mental Health Assessment Instruments: An Integrated System for the Continuum of Care. Front. Psychiatry. 2020; 10: 926. DOI: 10.3389/fpsyt.2019.00926 32076412 PMC6978285

[62] Longpré C, Dubois CA. Fostering development of nursing practices to support integrated care when implementing integrated care pathways: what levers to use? BMC health services research. 2017; 17(1): 790. DOI: 10.1186/s12913-017-2687-0 29187191 PMC5706162

[63] Janse B, Huijsman R, de Kuyper R, Fabbricotti I. Do integrated care structures foster processes of integration? A quasi-experimental study in frail elderly care from the professional perspective. International Journal for Quality in Health Care. 2016; 28(3): 376–383. DOI: 10.1093/intqhc/mzw045 27174858 PMC4931912

[64] Hartmann ML, Mello JDA, Anthierens S, Declercq A, Van Durme T, Ces S, Verhoeven V, Wens J, Macq J, Remmen R. Caring for a frail older person: the association between informal caregiver burden and being unsatisfied with support from family and friends. Age and Ageing. 2019; 48(5): 656–662. DOI: 10.1093/ageing/afz054 31147671

[65] Betini RSD, Hirdes JP, Curtin-Telegdi N, et al. Development and validation of a screener based on interRAI assessments to measure informal caregiver wellbeing in the community. BMC Geriatr. 2018; 18: 310. DOI: 10.1186/s12877-018-0986-x 30545318 PMC6293658

[66] Guthrie DM, Williams N, Beach C, et al. Development and Validation of Caregiver Risk Evaluation (CaRE): A New Algorithm to Screen for Caregiver Burden. Journal of Applied Gerontology. 2021; 40(7): 731–741. DOI: 10.1177/0733464820920102 32456510

